# Impact of COVID-19 Lockdown on Body Weight, Eating Habits, and Physical Activity of Jordanian Children and Adolescents

**DOI:** 10.1017/dmp.2021.48

**Published:** 2021-02-16

**Authors:** Huda Al Hourani, Buthaina Alkhatib, Mai Abdullah

**Affiliations:** 1 Department of Clinical Nutrition and Dietetics, Faculty of Applied Medical Sciences. The Hashemite University, Zarqa, Jordan; 2 Department of Clinical Nutrition and Dietetics, Faculty of Science, Philadelphia University; Amman, Jordan; 3 Department of Nutrition and Food Processing, Al-Huson University College, Al-Balqa Applied University, Jordan

**Keywords:** school children, weight gain, COVID-19, lockdown

## Abstract

**Objective:**

The lockdown for the COVID-19 pandemic affects lifestyle patterns globally and impacts children and adolescents. This study aims to assess the effect of the lockdown on body weight, eating habits, and physical activity of Jordanian youth (children and adolescents).

**Methods:**

A cross-sectional study was conducted on a sample of 477 Jordanian children and adolescents aged 6-17 y. The study tool was a structured validated questionnaire. It comprised 4 parts, including a general description of the study purpose, sociodemographic and anthropometric data, physical activity data, and food intake pattern. Questions were reported before and during lockdown. Changes in outcomes over the 2 study time points were evaluated.

**Results:**

After the lockdown period, the mean body weight and body mass index for age Z-scores (BAZ) showed a significant increase (*P* < 0.001) compared with before the lockdown period. More than 50% of the subjects reported that they spent more than 3 h in front of the screen during the lockdown. The percent of subjects who watched TV for more than 3h was increased. Moreover, physical inactivity was increased significantly during the lockdown. All food groups consumption was significantly increased during the lockdown compared to before the lockdown.

**Conclusions:**

COVID-19 lockdown period was characterized by an increase in the use of screen-based devices, lower physical activity, uncontrolled food intake, and weight gain.

The novel coronavirus disease 2019 (COVID-19), first reported in December 2019 in China, rapidly spread outside China and the Asian countries. On March 2020, it was declared a pandemic by the World Health Organization.^1,2^ Under these conditions, many countries were forced to adopt strict public health measures with the appearance of their first cases and the declaration of lockdown strategies at the local and international levels, to slow down the spread of COVID-19 pandemic. Following that was a forced quarantine at home to an estimated 4 billion people,^3^ which has had negative health impacts with substantial morbidity and mortality rates.^4^ Globally, the negative effects of the COVID-19 heath impacts expanded to include social and economic impacts, accompanied with sudden discontinuation of school programs for children and adolescents.

In Jordan, after recording the first case of COVID-19 among citizens, the government suspended schools, prohibited public gatherings, and closed borders and airports. For a period of 10 wk, the closure included a ban on the use of cars, with the exception of health providers and primary sector workers. Individuals only were allowed to move on foot during the daylight hours from 10:00 am in the morning until 6:00 pm in the evening.^5^ Children under 16 and older persons over 65 y of age were prohibited from leaving homes.^6^ The school year was continued through e-learning courses by means of distance learning system using electronic and TV screens.^7^

During the severe acute respiratory syndrome (SARS) pandemic, it was reported that there was a high prevalence of psychological stress, low mood, and irritability followed the quarantine^8^ associated with exhaustion and emotional disturbance, anger, insomnia, and depressive symptoms.^9-11^ Depression and stress push individuals to consume high sugar food and to choose high calorie foods to boost their mood.^12,13^ Prolonged staying at home may also to promote hypercaloric diets and more snacking; it may further affect individual choices to cook more or buy prepared food more often, and also may lead to unlimited access to food associated with low physical activity.^14-16^

Recently, Rundle and colleagues (2020) strengthened the argument that the COVID-19 pandemic, by preventing children from going to school, will enhance the “weight gain” factors associated with the summer vacation. The authors hypothesized that homes will be stored during closure with heavily processed and high energy-dense foods.^4^

Action plans on COVID-19 quarantine have been closely linked with obesogenic practices especially low physical activity, increased food consumption and unhealthy eating habits. The situation has been created where people are sitting in the same place for extended periods of time, enhancing sedentary behaviors, such as exposure to screens (computers, video games, tablets, television, smartphones), remote working for those people who can perform jobs from home and the closure of gardens, gyms, and sports spaces.^17^ Jakobsson et al. indicated that maintaining regular physical activity during self-isolation was important for weight gain prevention.^18^ A recent study of the Australian population indicated that 43.4% of the population exercised less during the COVID-19 pandemic, and this was correlated with binge eating and exercise.^19^ Moreover, the perception of body weight increasing during lockdown has been observed in 48.6% of the Italian population.^20^ Reyes-Olavarría and colleagues (2020) found that, during COVID-19 confinement, the body weight increase correlated with a positive association with the consumption of fried foods, low water consumption, and sedentary time ≥ 6 h/day. On the other hand, fish consumed, active breaks and physical activity ≥ 4 times per wk presented an inverse association with body weight increase.^17^

Children and adolescents (youth) represent a substantial proportion of the population of Jordan (30%).^21^ A cross-sectional study was conducted in Jordan by Zayed and colleagues in 2016 with 2702 subjects aged 6-17 y. It was reported that the prevalence of overweight and obesity was 17.3%, and 15.7%, respectively.^22^ In 2016, the overall prevalence of malnutrition characterized by children that were overweight and obesity aged 10-17 y old was 46.1% in greater Amman.^23^ A systematic study done by Ng and colleagues (2014) reported that the prevalence of overweight and obesity between boys and girls <20 y were 24.1% and 25.4%, respectively.^23-25^ Furthermore, Musaiger at al. (2016) highlighted the prevalence of overweight and obesity among adolescents in 8 Arab countries, in a school-based cross-sectional study with a sample size of 6447 adolescents aged 15-18 y. The overweight and obesity rate among Jordanian adolescents according to WHO reference standards were 11.8% and 10.6%, respectively.^25^ In light of this information, the aim is to study the effect of home quarantine, distance learning, and the lockdown on weight, eating habits, and physical activity of Jordanian children and adolescents under the age of 16 y(children and adolescents) for a period of 10 wk of quarantine during the COVID-19 pandemic.

## Methods

### Study Design

This is a cross-sectional study was conducted June 15-30, 2020, among 447 children and adolescents aged 6-17 y in Jordan. The calculated sample size was 384 based on the population size of the study. The study relied upon a structured validated questionnaire built by using Google Form. The questionnaire was sent to the mothers of the participants by means of WhatsApp and Facebook Messenger only using convenience and snowball sampling techniques, and parental consent/permission was obtained. Adolescents filled out the questionnaire themselves after receiving it from their mothers while their mothers filled out the children’s questionnaire; the interested mothers agreed to participate completing the questionnaire. The authors and the trained nutritionist provided assistance to the mothers or adolescents who had troubles filling out the questionnaire; in particular, the food consumption part. The food consumption part was illustrated by photos of the food items in the specified portion size. The questionnaire comprised 4 parts, including a general description of the study purpose and agreement of the respondent to participate, self-reported sociodemographic and anthropometric data, physical activity data, and food intake pattern. The questionnaire was designed to take 10-15 min to be completed. Informed consent for participation was obtained on the first page of the survey. Ethical approval for this study was approved by the Faculty of Science at Philadelphia University. The anonymous design of the Web survey will not allow sensitive personal data to be tracked in any way.

### Self-Reported Anthropometric Measurements

Participants self-reported their weight and height, and their body mass index (BMI) was calculated. Weight and height were entered into WHO Anthro-Plus software (v1.0.4, WHO, Geneva, Switzerland). BMI-for-age Z-scores (BAZ) and height-for-age Z-scores (HAZ) were calculated. For the association of the BMI-for-age with overweight and obesity, values > +1 standard deviation (SD) represent overweight (equivalent to BMI 25 kg/m^2^ at 19 y) and > +2 SD represent obesity (equivalent to BMI 30 kg/m^2^ at 19 y) according to the WHO reference curves (2007). Whereas, values between +1 SD and −2 SD was considered normal and values > −2 and −3 were considered thinness and severe thinness, respectively. Regarding HAZ, the children were classified into the following categories: stunting (low height-for-age < −2 *Z*-scores), < −3.00 were defined as severely stunted, and values above −2 SD were considered normal height.^26^

### Instruments

A self-administered semi-quantitative food frequency questionnaire (FFQ) was designed for this study to assess the consumption of selected food items. A pilot test was conducted on 40 subjects who were not included in the final sample. The 13- to 17-y-old participants or parents of 6- to 12-y-old participants were asked about the food items they or their children had consumed before and during the lockdown. Participants were instructed how to report the frequency and number of standard portions, which were consumed before and during the lockdown. An illustration was provided as an example to report 1 cup of milk was taken once daily in the lockdown time. The FFQ consisted of 47 food items; answer categories were “never,” “once weekly,” “2-4 times per week,” “5-6 times per week,” “once daily,” “2-3 times per day,” “more than 3 times per day.” Pictorial aid for fixed serving sizes of foods was included. The questionnaire included 8 food groups: milk and milk products (6 items), vegetables (2 items), fruits (4 items), bread, grains and pasta (9 items), protein rich foods (7 items), beverages (4 items), snack foods (6 items), and sweets and desserts (9 items). The Cronbach’s coefficient alpha, for reliability of the FFQ, ranged between 0.79 and 0.93.

Authors and a trained nutritionist helped with further questions regarding the completion of the questionnaire. **Supplementary Table** shows the list of foods under each of the food groups and the definition of serving size for each group.

Daily consumption of each FFQ food item in serving was calculated by converting the frequency of consumption into daily equivalents (never = 0; 1/wk = 0.14; 2-4/wk = 0.43; 5-6/wk = 0.8; 1/d = 1.0; 2-3/d = 2.5; ≥ 3/d = 3.0) and then multiplying by the calculated portion size for that food.^27^

### Statistical Analysis

Analyses were carried out using SPSS software (IBM SPSS Statistics for Windows, Version 22.0. Armonk, NY: IBM Corp). Continuous variables were described using means and SDs, and categorical variables were described using percentages. The chi-squared test was used to compare percentages. Paired t-test was used to compare variables at the before and after the lockdown. A *P*-value of < 0.05 was considered statistically significant.

## Results

### Sociodemographic Characteristics of the Participants

A total of 477 participants were included for the data analysis based on the number of returned valid questionnaires; ages were between 6 and 17 y. Participants were split into 2 groups: children aged 6-12 y (51.4%) and adolescents aged 13-17 y (48.6%). as shown in Table 1. More than half of the respondents lived in apartments (54.1%), and 43.4% had a family monthly income ranges between 500 and 1000 Jordanian Dinar.

**Table 1.** Sociodemographic characteristics of the participants

Characteristics	N (%)
**Gender**	
Males	231 (48.4)
Females	245 (51.6)
**Age group (y)**	
6-12	245 (51.4)
13-17	232 (48.6)
**Type of houses**	
Apartments	258 (54.1)
Single family (detached)	157 (32.9)
Family house	56 (11.7)
Others	6 (1.3)
**Family income (JOD/month)**	
Less than 500	129 (27.0)
500 – 1000	207 (43.4)
1000 – 1500	63 (13.2)
More than 1500	78 (16.4)

### Anthropometric and Physical Activity Characteristics of the Participants

Table 2 displays participant’s self-reported anthropometric and physical characteristics. As far as the Z-score values are concerned, the HAZ values showed that 86.9% of children and 93.9% of adolescents were of normal height and the rest were divided into 2 categories: stunting and severely stunting. In the children group (6-12 y), the overall prevalence of overweight and obesity before the lockdown was 18% and 16.7%, respectively, and both rose to 24.1% after the lockdown. On the other hand, the prevalence overweight and obesity for adolescents before the lockdown were 23.3% and 12.9%, respectively, while the prevalence of overweight (20.7%) was decreased and the prevalence of obesity (16.4%) was increased during the lockdown for adolescents. Severe thinness and thinness showed a substantial decrease in their proportion (*P* ≤ 0.001).

**Table 2.** Anthropometric and physical activity characteristics of the participants before and during lockdown time

Characteristics	6-12 y		*P*-Value	13-17 y		*P*-Value
	Before	During		Before	During	
Height (cm)^a^	135.3 ± 14.1			162.5 ± 11.1		
HAZ^b^						
Normal	213 (86.9)			217 (93.5)		
Stunted	18 (7.3)			9 (3.9)		
Severely stunted	14 (5.7)			6 (2.6)		
Weight (kg)^a^	33.4 ± 10.5	35.7 ± 11.5	< 0.001	58.1 ± 16.1	59.8 ± 16.7	< 0.001
BAZ^a^	0.32 ± 1.9	0.82 ± 1.9	< 0.001	0.35 ± 1.43	0.54 ± 1.47	< 0.001
BMI classification^b^						
Severe thinness	13 (5.3)	10 (4.1)	< 0.001	5 (2.2)	3 (1.3)	< 0.001
Thinness	13 (5.3)	7 (2.9)		6 (2.6)	6 (2.6)	
Normal	134 (54.7)	110 (44.9)		137 (59.1)	137 (59.1)	
Overweight	44 (18.0)	59 (24.1)		54 (23.3)	48 (20.7)	
Obese	41 (16.7)	59 (24.1)		30 (12.9)	38 (16.4)	
Studying^b^						
Less than 1 h	22 (9.0)	79 (32.2)	< 0.001	31 (13.4)	112 (48.3)	< 0.001
1 - 2 h	106 (43.3)	67 (27.3)		79 (34.1)	60 (25.9)	
2 - 3 h	85 (34.7)	56 (22.9)		72 (31.0)	19 (8.2)	
More than 3 h	32 (13.1)	43 (17.6)		50 (21.6)	41 (17.7)	
Screen time^b^						
Less than 1 h	83 (34.0)	39 (16.0)	< 0.001	37 (15.9)	13 (5.6)	< 0.001
1 - 2 hours	83 (34.0)	49 (20.1)		54 (23.3)	28 (12.1)	
2 - 3 h	39 (16.0)	52 (21.3)		43 (18.5)	25 (10.8)	
3 - 4 h	23 (9.4)	43 (17.6)		46 (19.8)	52 (22.4)	
More than 4 h	16 (6.6)	61 (25.0)		52 (22.4)	114 (49.1)	
Watching TV^b^						
Not watching TV	24 (9.8)	24 (9.8)	< 0.001	87 (37.5)	85 (86.3)	< 0.001
Less than 1 h	80 (32.7)	41 (16.7)		59 (25.4)	36 (15.5)	
1 - 2 h	99 (40.4)	69 (28.2)		51 (21.4)	39 (16.8)	
2 - 3 h	66 (10.6)	59 (24.1)		22 (9.5)	25 (10.8)	
3 - 4 h	12 (4.9)	27 (11.0)		4 (1.7)	26 (11.2)	
More than 4 h	4 (1.6)	25 (10.2)		4 (1.7)	21 (9.1)	
Having food while watching TV^b^						
Yes	117 (48.0)			106 (45.7)		
No	60 (24.6)			42 (18.1)		
Some times	68 (27.4)			84 (36.2)		
Playing time^b^						
None	41.(16.7)	73 (29.8)	< 0.001	44 (19.0)	99 (42.7)	
Less than 1 h	62 (25.3)	49 (20.0)		62 (26.7)	44 (19.0)	
1 h	63 (25.7)	41 (16.7)		50 (21.6)	36 (15.5)	
2 h	49 (20.0)	43 (17.6)		40 (17.2)	19 (8.2)	
≥ 3 h	30 (12.2)	39 (16.1)		36 (15.5)	34 (14.6)	

a Mean ± SD.

b *n* (%).

In both age groups, the hours spent studying were significantly lowered (*P* ≤ 0.001) compared with before the lockdown. About the subjects’ regular screen use time, it was found significantly that around of 70% of the adolescents reported spending more than 3 h in front of the screen during the lockdown, while it was 40% in the children group.

It was noticed that 86% of the adolescents did not watch TV during lockdown compared with 37.5% before lockdown, while the results showed no difference in children group. Almost 50% of both groups reportedly ate meals or snacks while watching TV.

Not unexpectedly, during the lockdown, physical inactivity increased dramatically, as more than 50% of subjects did not have physical activity or had less than 1 h of physical activity. Until the lockdown, walking was identified as the principal physical activity for them.

### Weight Gain Comparison Before and During the Lockdown

**Figure 1.**
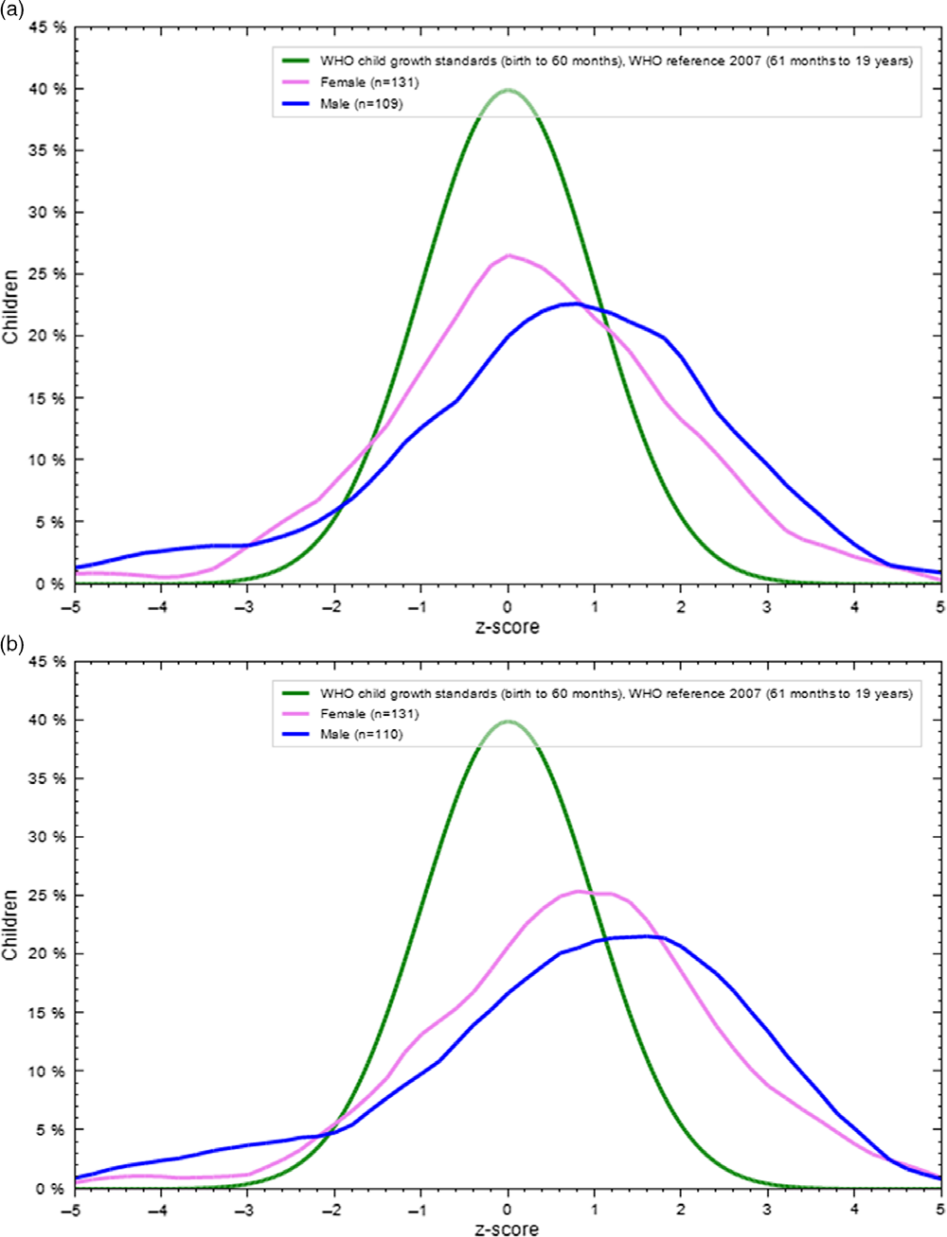
(a) BAZ distribution of children compared with the WHO reference curve before lockdown. (b) BAZ distribution of children compared with the WHO reference curve during lockdown.

**Figure 2.**
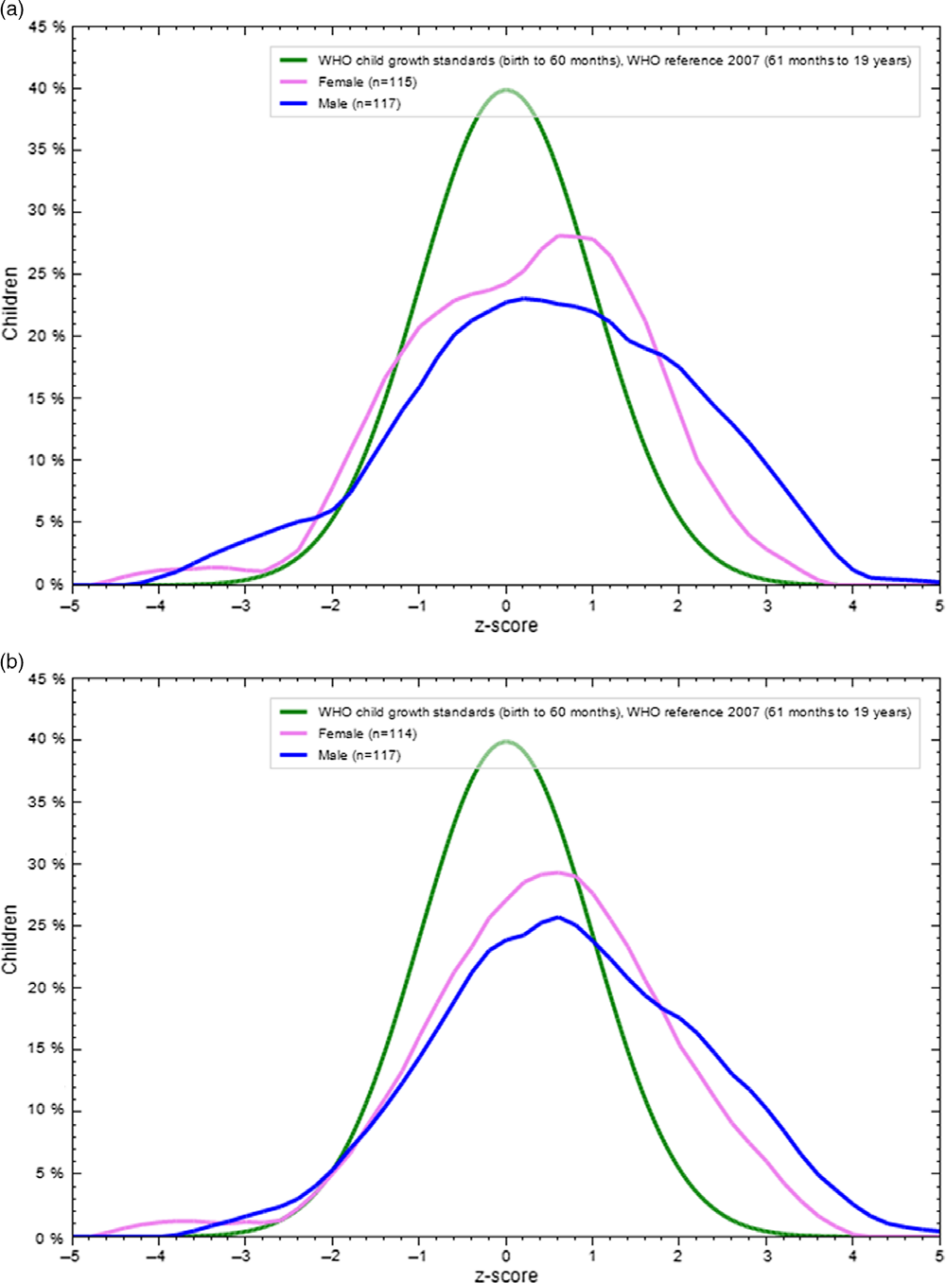
(a) BAZ distribution of adolescents compared with the WHO reference curve before lockdown. (b) BAZ distribution of adolescents compared with the WHO reference curve during lockdown.

The mean BMI-for-age Z score for children was 0.32 ± 1.9 before pandemic and 0.82 ± 1.9 during the lockdown, while 0.35 ± 1.43 before lockdown and 0.54 ± 1.47 in the adolescent group during the lockdown. The findings showed a substantial difference between the 2 mean BAZ (*P* < 0.001) as shown in Table 2. Also, **Figures 1** and **2** display the BAZ distribution of both age groups and genders before and during the pandemic, compared with the WHO reference curve. In children, BAZ showed a wide shift from WHO child growth standards for both genders during lockdown compared with before lockdown, which showed that the boys’ curve had only shifted away. In adolescents, BAZ showed a slight shift from WHO child growth standards in both genders and periods.

### Food Consumption Based on Food Groups

At a glance, children failed to meet recommended servings of fruits and meat, although **Table 3** revealed a significant rise in the consumption of all food groups during the lockdown compared with the pre-lockdown. The only exception to this was presented in some food items in the meat group. The most surprising result was the amount of bread consumed before and during the lockdown; bread is considered as the staple food in Jordan and it is offered in all main meals and snacks.

**Table 3.** Comparison of the intake of selected foods by diet groups (in servings per day) of the subjects before and during lockdown time

Foods (servings per day)	6-12 y		*P*-Value	13-17 y		*P*-Value
	Before	During		Before	During	
Milk and milk products, total						
Milk	4.0 ± 2.3	4.9 ± 2.9	<0.001	3.8 ± 2.7	4.2 ± 3.5	0.001
Labaneh	0.80 ± 0.72	0.86 ± 0.82	0.113	0.59 ± 0.60	0.65 ± 0.81	0.156
Creamy cheese	0.93 ± 0.77	1.04 ± 0.88	0.008	0.90 ± 0.83	1.0 ± 0.94	0.004
	0.73 ± 0.77	0.74 ± 0.84	0.648	0.68 ± 0.74	0.74 ± 0.90	0.064
Cooked vegetables	0.59 ± 0.59	0.64 ± 0.67	0.041	0.53 ± 0.67	0.63 ± 0.80	< 0.001
Raw vegetables	0.82 ± 0.64	0.92 ± 0.76	0.003	0.68 ± 0.65	0.76 ± 0.77	0.022
Fruits, total	2.8 ± 2.2	3.1 ± 2.5	<0.001	2.7 ± 2.9	3.2 ± 3.4	<0.001
Fresh fruits	1.3 ± 0.88	1.1 ± 0.94	0.001	1.1 ± 0.92	1.3 ± 1.1	<0.001
100% fruit juice	0.98 ± 1.2	1.1 ± 1.5	0.001	1.1 ± 1.6	1.3 ± 1.7	0.032
Breads, Grains, Pastas						
Bread	14.4 ± 9.2	15.2 ± 10.0	0.022	14.6 ± 11.4	16.4 ± 13.2	<0.001
Rice	0.93 ± 0.61	1.0 ± 0.62	<0.001	0.93 ± 0.59	1.0 ± 0.73	0.002
Macaroni and pasta	0.63 ± 0.99	0.67 ± 1.07	0.273	0.48 ± 0.72	0.53 ± 0.80	0.161
Cornflakes	0.99 ± 1.03	1.01 ± 1.44	0.687	0.85 ± 1.3	0.98 ± 1.6	0.048
Meat, poultry, fish and eggs						
Red meat	0.94 ± 1.2	1.01 ± 1.4	0.03	1.2 ± 1.6	1.2 ± 1.8	0.558
Chicken	0.71 ± 0.51	0.78 ± 0.61	0.003	0.80 ± 0.60	0.83 ± 0.70	0.331
Fish	0.26 ± 0.49	0.27 ± 0.53	0.501	0.28 ± 0.60	0.25 ± 0.58	0.402
Boiled eggs	0.55 ± 0.59	0.62 ± 0.68	0.011	0.54 ± 0.65	0.59 ± 0.74	0.029
Beverages						
Carbonated beverages	0.52 ± 0.74	0.64 ± 0.91	<0.001	0.70 ± 0.85	0.82 ± 1.0	0.005
Canned juice	0.82 ± 0.82	0.86 ± 0.94	0.211	0.70 ± 0.81	0.79 ± 0.96	0.027
Tea	0.76 ± 0.82	0.93 ± 0.96	<0.001	0.81 ± 0.86	1.0 ± 1.0	<0.001
Coffee	0.20 ± 0.52	0.22 ± 0.59	0.088	0.30 ± 0.60	0.36 ± 0.70	0.007
Snack foods						
French fries	0.58 ± 0.68	0.68 ± 0.78	0.001	0.53 ± 0.65	0.67 ± 0.80	<0.001
Pastries (Fatayer)	0.35 ± 0.54	0.41 ± 0.68	0.05	0.35 ± 0.61	0.45 ± 0.78	0.002
Pizza	0.36 ± 0.66	0.36 ± 0.65	0.992	0.34 ± 0.63	0.41 ± 0.76	0.001
Potato chips	1.1 ± 0.79	1.2 ± 0.91	0.174	1.1 ± 0.86	1.3 ± 1.0	<0.001
Popcorn	0.48 ± 0.68	0.58 ± 0.77	0.003	0.46 ± 0.63	0.59 ± 0.77	<0.001
Sweets and desserts						
Sugar	1.1 ± 0.0.95	1.2 ± 0.1.0	0.001	1.1 ± 1.0	1.3 ± 1.1	<0.001
Ice-cream	0.74 ± 0.66	0.89 ± 0.77	<0.001	0.81 ± 0.79	0.96 ± 0.92	<0.001
Cake	0.44 ± 0.59	0.56 ± 0.77	<0.001	0.49 ± 0.75	0.56 ± 0.83	0.063
Chocolate bar	0.93 ± 0.67	1.1 ± 0.86	<0.001	1.0 ± 0.86	1.2 ± 0.99	0.008
Arabic sweets	0.29 ± 0.50	0.37 ± 0.68	0.001	0.36 ± 0.67	0.44 ± 0.78	0.016

## Discussion

Globally, the outbreak of COVID-19 has forced different countries to implement strict social distancing measures and sanitary regimens. People were under the lockdown, working remotely, home schooling children, and facing quarantine challenges related to eating habits, sleeping time, physical inactivity, and stress. Also, COVID-19 quarantine led to failure to comply with positive eating habits, enhancing negative obesogenic eating habits.

Many studies have relied on comparing the quarantine situation with the students’ school vacation, whether in winter or summer vacation. Not surprisingly, this study’s results demonstrated a significant body weight and BMI increase in children and adolescents after the lockdown period (10 wk), which is in line with the results of previous studies on the effect of holidays, summer vacations, or disease pandemic curfew on weight gain in school children and adults.^3,14,28-35^

In this study, children, BAZ showed a wide shift from normal to overweight and obesity for both genders during lockdown compared with before lockdown, while in adolescents, BAZ showed a slight shift from WHO child growth standards in both genders and age periods. Branscum and colleagues (2010) found that the children gained 0.56 kg and raised their BMI by 0.28 kg/m^2^ during the holidays.^36^ The findings of the current research are close to those reported in the Schoeller (2014) literature review, which described a weight gain of approximately 0.5 kg.^37^ Furthermore, Moreno et al. (2013) found that all children after 1st grade in the summer demonstrated an increasing in pattern of standardized BMI (zBMI) (0.04 to 0.09) and zBMI decreasing across the school years (20.06 to 0.00; *P* < 0.0001).^31^

Recently, Zachary et al., (2020) also quantified the impact that self-quarantine on behaviors associated with weight gain by introducing a Facebook survey. They found that 91% percent of the respondents stated they were spending more time at home than they were before the COVID-19 lockdown. Around 22% of the sample reported gaining 5-10 pounds.^38^ Of interest, this study revealed a significant decrease in severe thinness, thinness, and normal weight percent during the lockdown compared with BMI before the lockdown. In contrast, overweight and obese percentages were increased significantly after the lockdown compared with before the lockdown. A narrative review conducted in 2014 by Baranowski and colleagues revealed that overweight and obese children had accelerated weight gain during the summer compared with those of normal weight.^34^ In 2013, Stevenson et al.^30^ found that participants significantly gained 0.78 kg (*P* < 0.001) and increased their fat percentage significantly by 0.5% (*P* = 0.007) during the Christmas period. It was observed that overweight and obese children increased their BMI percentile and weight more than children of normal weight during holiday,^36^ in contrast to underweight children who tended to lose weight further during the COVID-19 lockdown.^14^

In Australia, Phillipou et al. in 2020 evaluated the changes in weight and dietary habits in a sample of 150 obese outpatients after 1 mo of enforced lockdown during the COVID-19 pandemic. They found that higher weight gain was highly correlated with low exercise, self-reported boredom/isolation, anxiety/depression, enhanced eating, consumption of snacks, unhealthy foods, cereals, and sweets.^19^

As a result of the unusual events of forced isolation or quarantine during the COVID-19 semi-lockdown in China, He et al., (2020) found that both females and males with BMI < 24 gained weight, males with BMI ≥ 24 lost weight, compared with females with BMI ≥ 24 who gained weight. The average amount of exercise declined significantly for both genders during the semi-lockdown. Moreover, changes in body weight inversely correlated with changes in physical activity during the quarantine.^39^

In the following parts, this study will focus on weight gain and obesogenic behaviors (excess food consumption, physical activity, and/or sedentary behaviors) associated with the lockdown. Excess screen use is 1 of the sedentary behaviors; the present data showed that daily screen use time for more than 3 h was significantly noticed in more than 50% of the participants during the lockdown, compared with 50% spent less than 2 h before the lockdown. Almost average of 47% of the total participants reportedly ate meals or snacks while watching TV. Moreover, playing time was decreased significantly, the percent of participants who do not play increased from 16.7% before the lockdown to 29.8% after the lockdown for children, from 19% to 42.7%, respectively, for adolescents. Supporting these findings, data from U.S. children in grades 1-12 in the National Health and Nutrition Examination Survey 2003-2008, revealed that children watched more television during summer breaks (+18 min/d).^28^ Olds and colleagues (2019) found that on holidays, children accumulated 140 min less school-related time, compensated by sleeping 40 min longer, 58 min more screen time, and 35 min more domestic/social time, children spent 10 min less in vigorous physical activity.^35^ Oppositely, Zinkel et al. (2013) reported that there was no substantial change in total energy expenditure (adjusted for fat-free mass) and in the level of physical activity in summer versus in school months among children from the United States.^32^ Robinson et al. (2017) explored the mechanisms behind the effect of screen use on obesity, which included displacing physical activity, increase food intake while viewing, effect of food advertising, and reducing sleeping hours.^40^

In their investigation during COVID-19 lockdown, Di Renzo and colleagues (2020) observed the perception of weight gain in 48.6% of the population study in the North and Center of Italy; the population group aged 18-30 y resulted in having a higher adherence to the Mediterranean diet when compared with the younger and the elderly population; 15% of respondents turned to farmers or organic, purchasing fruits and vegetables, where BMI values were lower.^20^

The other obesogenic factor, which was highlighted in this study, was excess food intake. The present study’s results showed almost a significantly increased intake of all food groups during the lockdown compared with before the lockdown. The only exception was presented in some food items in meat group. Although that increased, children failed to meet recommended servings of vegetables. It is striking to take into consideration some traditional nutritional habits in Jordan, like the availability of several types of bread and eating sandwiches and wheat baked goods during the day as snack, which increases the daily amount of bread intake. Bracale and colleagues (2020) have demonstrated an increase in pasta, flour, eggs, long shelf-life milk, and frozen foods consumption compared with decreased consumption of fresh food during COVID-19 lockdown. Moreover, snack sales have plummeted in relation to the production of homemade bread, pizza, and cakes.^41^ Also, Zachary and colleagues found that participants who gained weight during COVID-19 reported significant increased eating in response to sight and smell (*P* = 0.048), eating in response to stress (*P* = 0.041), and snacking after dinner (*P* = 0.016) compared with those who stated they did not change those behaviors at all.^38^ In their investigation of the impact of COVID-19 lockdown on the body weight of 700 children, their eating pattern, and physical activity, Reyes-Olavarría and colleagues (2020) found that the body weight increase correlated with positive association with the consumption of fried foods ≥ 3 times per wk, low water consumption, and sedentary time ≥ 6 h/day. While physical activity ≥ 4 times per wk presented an inverse association with body weight increase.^17^

During the lockdown of COVID-19 quarantine, Sidor and Rzymski (2020) found that over 43.0% and nearly 52% reported eating and snacking more, respectively. In addition, less frequent consumption of vegetables, fruit, and legumes during quarantine and higher adherence to meat, dairy, and fast-foods associated with increasing BMI.^14^ Concomitantly, Pietrobelli and colleagues (2020) found that the intake of potato chips, red meat, and sugary drinks increased significantly during the lockdown, while the time spent in sports activities decreased by 2.30 ± 4.60 h/wk (*P* = 0.003), and sleeping and screen time were significantly increased by 0.65 ± 1.29 h/day and 4.85 ± 2.40 h/day, respectively. They reported that there were no significant changes in the increase of vegetable and fruit intake (*P* = 0.055).^3^ In an international online survey, which was launched in April 2020, to elucidate the behavioral and lifestyle consequences of COVID-19 restrictions by 35 research organizations from Europe, North-Africa, Western Asia, and the Americas, researchers reported that COVID-19 home quarantine had a negative effect on all physical activity intensity levels. Additionally, daily sitting time increased from 5 to 8 h/day. Food consumption and meal patterns (the type of food, eating out of control, snacks between meals, number of main meals) were unhealthier during lockdown.^42^

Another concern should be posed in this current study, was the coincident event of the Holy month of Ramadan with the period of the lockdown. It is common with Jordanians to find some who practice fasting at 6 or 8 y of age. A limited number of studies discussed the effect of Ramadan fasting on body weight among children and adolescents. Two studies reported a weight loss among children and adolescents during Ramadan fasting.^43,44^ In contrast to previous studies, participants of the current study did not experience weight loss during the Holy month of Ramadan. This may be attributed to different obesogenic factors; especially the shift in eating time, where individuals eat large quantities of food and sweets during the night hours, and change sleeping habits to become a long sleep during the daylight hours and waking up at night hours. In any case, this current study’s data need to be confirmed and investigated in the future to determine the impact of these factors.

## Limitations

The current study had several limitations that should be acknowledged. The cross-sectional nature of the study precludes causality judgment between food intake and weight gain. Also, FFQ is not optimal for the measurement of absolute dietary intake. Additionally, lack of standardization of anthropometric measurements, the assessment of physical activity and food intake, as those were focused on self-reports, which were subject to recall and prejudice in social desirability. Sleeping hours was not recorded in the survey. There is a high possibility of volunteer bias among the study participants who have responded and completed the questionnaire when compared with the nonresponders. Finally, the convenience sampling, noninclusion of rural children, and including only those children who have access to technology needs to be considered while interpreting the study findings.

## Conclusions

In conclusion, COVID-19 lockdown affects the lifestyle of children and adolescents. This study concluded that food consumption was increased, physical activity was decreased, accompanied with an increase in screen time. School closing prompted an alarming shift in eating habits, amount of nutritional intake, weight gain, and physical inactivity. Therefore, children and adolescents are vital target groups, and the results warrant more future research to design plans and programs to avoid the negative vacation consequences related to overweight and obesity in children and adolescents, resulted from different obesogenic factors during COVID lockdown restriction.

## Supplementary material

For supplementary material accompanying this paper visit http://dx.doi.org/10.1017/dmp.2021.48. click here to view supplementary material
