# Supplementary material for: Impact of COVID-19 Lockdown on Body Weight, Eating Habits, and Physical Activity of Jordanian Children and Adolescents
Source: Disaster Med Public Health Prep. 2021 Feb 16:1–9. doi: 10.1017/dmp.2021.48 (PMC8129676; doi:10.1017/dmp.2021.48)
Supplement: Supplementary file 1 [file S1935789321000483sup001.docx]

**Supplementary Table**

| **Food Group** | **Foods** | **SERVING SIZE** |
| --- | --- | --- |
| Milk and milk products | Milk, yoghurt, white cheese in brine, spreadable processed cheese, cheddar flavored processed cheese, and labneh (condensed salted yoghurt) | One cup milk or yoghurt, 45gm of cheese, two tablespoons of labaneh. |
| Vegetables | Cooked vegetables such as green beans, okra, and spinach. Raw vegetables such as tomatoes, cucumber, lettuce, and cabbage | ½ cup cooked vegetables or one cup raw vegetables. |
| Fruits | Fresh fruits such as apples, banana, oranges. 100% fruit juices, dried and canned fruits | One small fresh size fruit, ½ cup 100% fruit juice, two tablespoons dried fruits, ½ cup canned fruits. |
| Bread, grains and cereals | Thin white or whole wheat Kmaj bread, thick Kmaj white or whole wheat bread, burger bun, Sammoun (Sammoli or Hamam, A pan white French- like bread), shrak bread (non- pocket, one-layered flatbread). Rice and rice in dishes, macaroni, cornflakes  [Kmaj bread is flat and round, one loaf measures about 15-25 cm in diameter; moisture content ranges from 25% to 38 %, and presented into two forms thin and thick]. | One ounce of bread (any type), ½ cup cooked rice or macaroni, ½ cup cornflakes |
| Meat, poultry, fish and eggs | Lamb and veal meat, chicken, fish, tuna and sardine, sausages, boiled and fried eggs, | 2-3 oz of cooked lamb, veal, chicken or fish, one small can of tuna or sardine, 2 egg, |
| Beverages | Carbonated beverages, canned juices (fruit drinks), tea, coffee | One cup carbonated beverages or canned juice, small cup (120 ml) of tea, one small cup of coffee (90ml) |
| Snack foods | French fries, Pastries (Fatayer), Rusk (**biscote**), potato chips, popcorn, pizza | Three ounces French fries, 60 gm fatayer? one-piece (1 oz) rusk, 1.5-ounce potato chips (one pack), three cup popcorn, four ounces pizza |
| Sweets and desserts | Sugar, jam and honey, ice cream, Jell-O, pudding, custard, chocolate, cake, Arabic sweets (baklava, harissa) | One teaspoon sugar, One Tablespoon jam or honey, ½ cup ice cream, ½ cup Jell-O or pudding or custard, one ounce of chocolate, two ounces cake, one ounce of Arabic sweets |

One cup equals 240 milliliters
